# Selected Phytochemicals and Culinary Plant Extracts Inhibit Fructose Uptake in Caco-2 Cells

**DOI:** 10.3390/molecules200917393

**Published:** 2015-09-18

**Authors:** Yurim Lee, Yeni Lim, Oran Kwon

**Affiliations:** Department of Nutritional Science and Food Management, Ewha Womans University, 52 Ewhayeodae-gil, Seodaemun-gu, Seoul 120-750, Korea; E-Mails: iamyourim@naver.com (Y.L.); yeni0223@hanmail.net (Y.L.)

**Keywords:** Caco-2 cells, fructose transport, culinary plants, GLUT2, GLUT5

## Abstract

This study compared the ability of nine culinary plant extracts containing a wide array of phytochemicals to inhibit fructose uptake and then explored the involvement of intestinal fructose transporters and phytochemicals for selected samples. The chemical signature was characterized by high performance liquid chromatography with mass spectrometry. Inhibition of [^14^C]-fructose uptake was tested by using human intestinal Caco-2 cells. Then, the relative contribution of the two apical-facing intestinal fructose transporters, GLUT2 and GLUT5, and the signature components for fructose uptake inhibition was confirmed in naive, phloretin-treated and forskolin-treated Caco-2 cells. HPLC/MS analysis of the chemical signature revealed that guava leaf contained quercetin and catechin, and turmeric contained curcumin, bisdemethoxycurcumin and dimethoxycurcumin. Similar inhibition of fructose uptake (by ~50%) was observed with guava leaf and turmeric in Caco-2 cells, but with a higher contribution of GLUT2 for turmeric and that of GLUT5 for guava leaf. The data suggested that, in turmeric, demethoxycurcumin specifically contributed to GLUT2-mediated fructose uptake inhibition, and curcumin did the same to GLUT5-mediated fructose uptake inhibition, but GLUT2 inhibition was more potent. By contrast, in guava leaf, catechin specifically contributed to GLUT5-mediated fructose uptake inhibition, and quercetin affected both GLUT5- and GLUT2-mediated fructose uptake inhibition, resulting in the higher contribution of GLUT5. These results suggest that demethoxycurcumin is an important contributor to GLUT2-mediated fructose uptake inhibition for turmeric extract, and catechin is the same to GLUT5-mediated fructose uptake inhibition for guava leaf extract. Quercetin, curcumin and bisdemethoxycurcumin contributed to both GLUT5- and GLUT2-mediated fructose uptake inhibition, but the contribution to GLUT5 inhibition was higher than the contribution to GLUT2 inhibition.

## 1. Introduction

Accumulating evidence indicates that high fructose consumption leads to the development of obesity, diabetes and dyslipidemia in experimental animals. Unlike glucose, absorbed fructose is nearly completely metabolized in the liver through the sequential actions of fructokinase, aldolase B and triokinase, which are not inhibited by ADP and citrate [1]. Due to this absence of feedback inhibition, almost all fructose absorbed is converted into hepatic triose-phosphate regardless of the cellular energy status [2]. Then, the resulting metabolites are disposed through various pathways, such as oxidation, conversion into glucose and lactose for release into the blood stream, conversion into hepatic glycogen or *de novo* lipogenesis [3]. In particular, increased consumption of fructose in the form of the disaccharide sucrose or high-fructose corn syrup (HFCS) has been implicated in the adverse metabolic effects of fructose [4]. In contrast, there is compelling evidence that consumption of fructose with fruits or honey does not yield the same unfavorable effects as added fructose, probably due to the presence of dietary fibers and/or phytochemicals.

In our previous study [5], we demonstrated that selected individual phytochemicals widely distributed in culinary plants inhibited fructose intestinal transport and absorption. This finding suggests that a new prevention option for obesity, diabetes and dyslipidemia could be based on strategies to dampen or inhibit fructose absorption. Moreover, it is generally assumed that an array of phytochemicals as existing in culinary plants may play additive or synergistic roles, providing more potent health benefits compared to an individual component [6]. However, little is known regarding culinary plant extracts as inhibitors specific for intestinal fructose absorption. The facilitative glucose transporters (GLUTs) mediate intestinal fructose transport from the intestinal lumen into enterocytes: GLUT2 transports both glucose and fructose, and GLUT5 transports fructose only. Then, both glucose and fructose exit enterocytes via GLUT2 [7]. The apical GLUT2 pathway may be particularly important postprandially, because it is regulated by rapid trafficking of GLUT2 to the apical membrane within minutes during assimilation of meals or foods containing high sugars [8]. Therefore, the two apical-facing facilitative fructose transporters, GLUT2 and GLUT5, are attractive targets for such potential phytochemicals [5].

In the present study, we compared the ability of commercially available culinary plant extracts to inhibit fructose uptake by human intestinal epithelial (Caco-2) cells. Then, for the selected samples, chemical signatures were identified by using high performance liquid chromatography (HPLC) with mass spectrometry (MS). Furthermore, an attempt was made to determine the relative contribution of intestinal fructose transporter GLUT2 and GLUT5 to fructose uptake inhibition by culinary plant extracts and their signature components by using phloretin- or forskolin-treated Caco-2 cells.

## 2. Results and Discussion

### 2.1. Cell Viability

**Figure 1:**
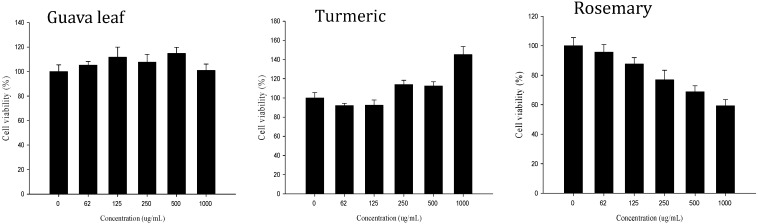
Effects of guava leaf, turmeric, and rosemary extracts on Caco-2 cell viability. Cells were incubated at different concentrations (0–1000 μg/mL) of culinary plant extracts for 1 h, and cell viability (as % of control) was determined by using the MTT assay. Values represent the mean ± SE.

The cytotoxicities of nine samples (extracts of guava leaf, turmeric, onion, Korean red ginseng, chrysanthemum, passion flower, touchi, rosemary and bayberry) were examined quantitatively at the range of 0–1000 μg/mL by the MTT assay (Figure 1). Cell viabilities were found to be unchanged for most samples with the exceptions of bayberry bark and rosemary extracts. Based on these results, the maximum concentration for all samples was chosen to test the ability of inhibiting fructose uptake.

### 2.2. Comparison of Fructose Uptake Inhibition

**Figure 2:**
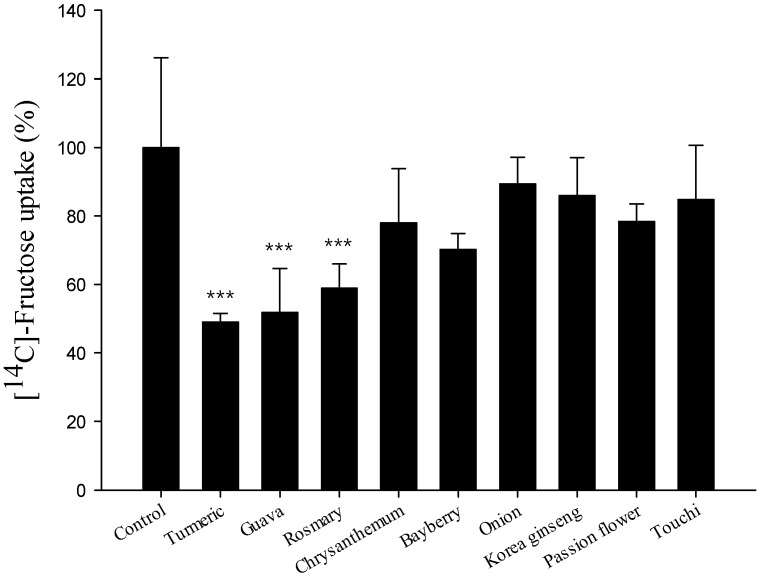
Comparison of the inhibitory effects of nine culinary plant extracts on fructose transport in Caco-2 cells. Cells were incubated in Krebs buffer containing 10 mM of [^14^C] fructose and 500 μg/mL concentrations of each sample for 10 min at 37 °C. Transport was determined by scintillation spectrometry. Values represent the mean ± SE. *** *p* < 0.0001 *vs.* the vehicle-treated control.

Inhibitory effects on fructose uptake were compared as a function of culinary plants at fixed concentrations. The fructose concentration was set at 10 mM, considering the maximum possible plasma concentration at postprandial condition; and the sample concentration was set at 500 μg/mL, as determined by the half of its maximum safe concentration in the MTT assay. Turmeric, guava leaf and rosemary extracts showed a significant inhibition of fructose uptake by ~50% (*p* < 0.0001) and, thus, were initially selected as the most potent inhibitors (Figure 2). Based on the findings of either the cell viability or the fructose uptake inhibition, we decided to undertake further investigation with turmeric and guava leaf extracts.

### 2.3. Chemical Signature of Culinary Plant Extracts

**Figure 3:**
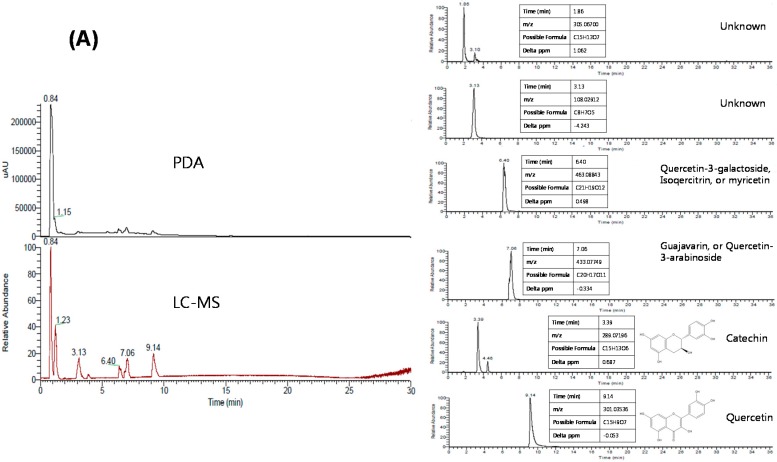

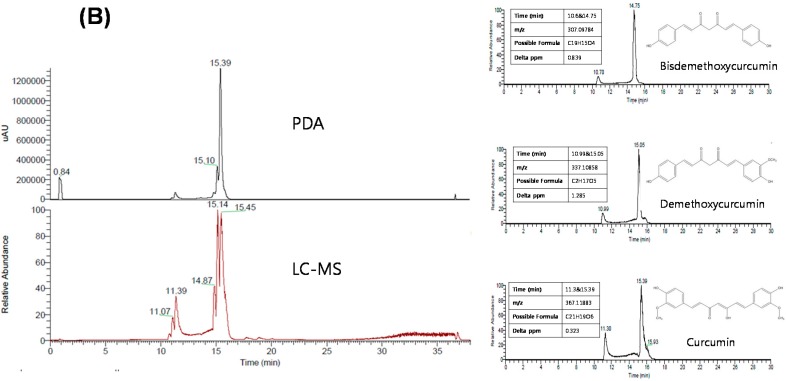
Simultaneous HPLC-PDA and HPLC-MS chromatograms of the guava leaf extract (**A**) and turmeric extract (**B**).

The simultaneous HPLC-PDA chromatogram and the HPLC-MS chromatogram of the guava leaf and turmeric extracts are presented in Figure 3. The best separation was observed with a reversed-phase Hypersil C18 column using gradient elution over 32 min at a flow rate of 0.3 mL/min. Acetonitrile was preferred as the mobile phase, and formic acid was added to the mobile phase for improved separation. HPLC-MS was used to identify the six peaks at retention times of 1.86, 3.13, 3.39, 6.40, 7.06 and 9.14 min in the chromatogram of guava leaf extract and three peaks at retention times of 14.75, 15.05 and 15.39 min in the chromatogram of turmeric extract. Comparison of the MS fragments and retention time of each corresponding peak confirmed the identification of quercetin-3-galactoside (or isoquercitrin, myricetin) at 6.40 min, quercetin-3-arabinoside (or guajavarin) at 7.06 min, catechin at 3.39 min and quercetin at 9.14 min from guava leaf extract and bisdemethoxycurcumin at 14.75 min, demethoxycurcumin at 15.05 min and curcumin at 15.39 min from turmeric extract. However, the other three components from guava leaf extract could not be identified due to the limited resource of information.

### 2.4. Contribution of GLUT2 and GLUT5 to Fructose Uptake Inhibition of Guava Leaf and Turmeric Extracts

**Figure 4:**
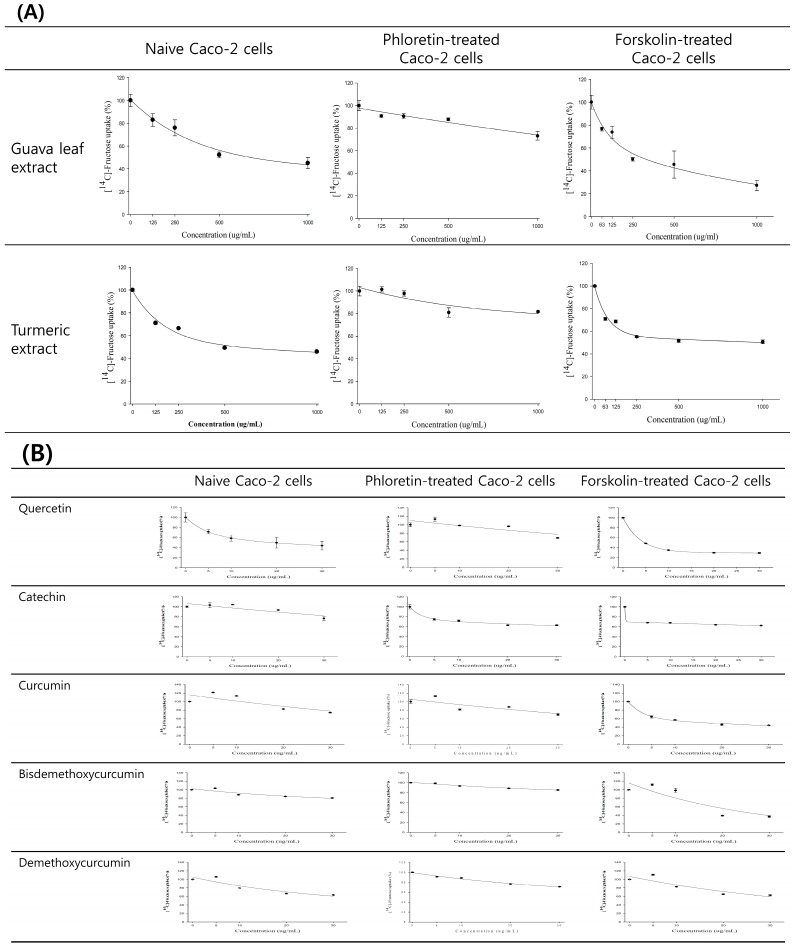
Inhibition of fructose uptake by guava leaf extract and turmeric extract (**A**) and their signature components (**B**) in naive, phloretin-treated and forskolin-treated Caco-2 cells. Cells were incubated in 10 mM [^14^C] fructose with increasing concentrations of samples for 10 min. Transport was determined by scintillation spectrometry. Values represent the mean ± SE.

The inhibitory effects of guava leaf and turmeric extracts on fructose uptake were compared at increasing concentrations (0–1000 μg/mL) in naive, forskolin (FK)-treated and phloretin-treated Caco-2 cells (Figure 4). In naive Caco-2 cells, fructose uptake was inhibited in a concentration-dependent manner up to 55.0 ± 4.9% by guava leaf extract and up to 54.1 ± 0.6% by turmeric extract. Phloretin treatment reduced GLUT2-mediated fructose transport, so that fructose uptake was reduced to 26.8 ± 3.8% by guava leaf extract and 18.3 ± 0.6% for turmeric extract, respectively. Changes in fructose uptake inhibition with and without phloretin were bigger in turmeric extract than in guava leaf extract. Interestingly, the pattern was reversed when Caco-2 cells were pretreated with FK; stronger inhibition was found for guava leaf extract than for turmeric extract. Taken together, these results suggested that GLUT2 provided a higher contribution to fructose uptake inhibition than GLUT5 for turmeric extract, while *vice versa* for guava leaf extract (Figure 4A).

Next, we compared the inhibitory effects of five signature components at increasing concentrations (0–30 μg/mL) in various cell systems. The pattern of inhibition and estimated IC_50_ values are presented in Figure 4B and Table 1, respectively. The rank of potency in Caco-2 cells was quercetin > demethoxycurcumin > curcumin > bisdemethoxycurcumin > catechin. When phloretin was used to treat Caco-2 cells to inhibit GLUT2-mediated fructose inhibition, the IC_50_ values were dramatically increased for bisdemethoxycurcumin and demothoxycurcumin, moderately increased for quercetin and not increased for catechin and curcumin. By contrast, when the GLUT5 gene was transiently overexpressed in Caco-2 cells by FK, the IC_50_ values were generally decreased, except for demethoxycurcumin. These results suggested that demethoxycurcumin is a specific contributor to GLUT2-mediated fructose uptake inhibition; catechin and curcumin are specific contributors to GLUT5-mediated fructose uptake inhibition; and quercetin contributed to both GLUT5- and GLUT2-mediated fructose uptake inhibition, but the contribution to GLUT5 inhibition was higher than the contribution to GLUT2 inhibition.

**Table 1:** IC_50_ values (μg/mL) for the inhibition of fructose uptake of five signature components in naive, phloretin-treated and forskolin-treated Caco-2 cells.

Samples	Caco-2 Cells	Phloretin-Treated Caco-2 Cells	Forskolin-Treated Caco-2 Cells
Quercetin	29.64	68.38	4.71
Catechin	87.08	63.75	37.72
Curcumin	65.57	58.86	16.84
Bisdemethoxycurcumin	83.60	NA	23.28
Demethoxycurcumin	37.43	NA	39.87

NA: not available.

### 2.5. Discussion

The hypothesis of this study was that culinary plants containing different compositions of phytochemicals might have different functions in dampening or suppressing fructose uptake from the intestinal lumen. Since Caco-2 cells have been widely employed to study intestinal nutrient transport [5,9], we have used them to test this hypothesis and measured the changes in fructose uptake at the apical side of intestinal cells. In the present study, guava leaf and turmeric extracts are preferentially selected, supporting the hypothesis. In the previous studies, others have shown that guava leaf extract modulated glucose uptake in rat hepatocytes [10], improved insulin resistance in skeletal muscle of rats with spontaneous metabolic syndrome [11], exerted an antihyperglycemic effect through antioxidative activity [12] and altered glucose metabolism in streptozotocin-induced diabetic rats [13]. In contrast, most research on turmeric extracts has been focused on the antioxidant defense system [14,15], prevention of the secondary complication of diabetes [14,16] and anti-inflammatory activities [17,18]. Taken together, these data provide additional possibilities of using guava leaf and turmeric extracts as a new prevention option for the risk of obesity, diabetes or dyslipidemia from excessive fructose intake.

Dietary fructose that is associated with metabolic defects can upregulate its absorption. When higher concentrations of fructose exist in the intestinal lumen, absorptive capacity increases in the small intestine according to the activity level, location within the enterocyte and gene expression [5,19]. GLUT5 and GLUT2 are the two primary transporters responsible for facilitative absorption of fructose. Therefore, here, using naive, phloretin-treated (GLUT2-inhibition) and FK-treated (GLUT5-overexpressing) Caco-2 cells, we demonstrated the relative importance of these two intestinal fructose transporters in inhibiting fructose transport by guava leaf and turmeric extracts. Guava leaf extract and turmeric extract showed similar potency to each other in Caco-2 cells. However, the inhibitory effect of guava leaf extract became stronger in GLUT5-overexpressing cells compared to naive Caco-2 cells. Interestingly, for turmeric extract, the higher reduction in fructose uptake inhibition was found in phloretin-treated Caco-2 cells, while no change was found in GLUT5-overexpressing cells. These data implied the different modes of action by which guava leaf and turmeric extracts might exert fructose transport inhibition. This triggered us to investigate the role of the phytochemicals contained in guava leaf and turmeric extracts.

HPLC/MS analysis revealed that guava leaf extracts contained quercetin and catechin as signature components, which belong to a group of polyphenolic substances known as flavonoids. In our previous study [5], a robust inhibition of fructose transport was observed by quercetin in GLUT2-injected oocytes, whereas no inhibition was found by catechin. Consistent with this result, by comparing fructose uptake inhibition in naive and phloretin-treated Caco-2 cells, we could confirm the previous result that catechin did not play a role in fructose uptake by GLUT2 inhibition. Instead, it inhibited fructose uptake by influencing GLUT5. We also found that quercetin had greater potency in fructose uptake inhibition than catechin. Both GLUT2 and GLUT5 were identified to be involved in quercetin inhibition of fructose uptake. On the other hand, HPLC/MS analysis also revealed that turmeric extract contained curcumin, bisdemethoxycurcumin and demethoxycurcumin as signature components, which belong to alkaloids. Demethoxycurcumin had the greatest potency among three components for fructose uptake inhibition through GLUT2 inhibition. The other two components, curcumin and bisdemethoxycurcumin, influenced both GLUT2 and GLUT5 for fructose uptake inhibition.

This is the first investigation to demonstrate the effects of guava leaf and turmeric extracts on fructose transport inhibition and to deal with exploring the action mechanism. The data obtained in this study clearly indicated that different components in each extract have different contributions to fructose transport inhibition by influencing GLUT2 or GLUT5 at the apical side of cells, suggesting a possibility that guava leaf and turmeric extracts might be useful to reduce the risks of obesity, diabetes or dyslipidemia from excessive fructose intake. The limitation of this study is that signature components of each extract were not quantified in the concentration per unit-gram. Therefore, the synergistic or antagonistic effects of each component on fructose uptake inhibition were not estimated. Another limitation is that the current study was incomplete to explain GLUT2- and GLUT5-trafficking at apical membranes. In addition, the fructose transport inhibition was not estimated at the basolateral side of cells, because we were using Caco-2 cells plated onto collagen-coated dishes to allow attachment. If components of culinary plant extracts can move across from the apical side into the cells, they can consequently inhibit the GLUT2 on the basolateral side of the cells [20]. Further study is needed to determine whether each component is transported into the cells and whether an array of components in culinary plant extract can give a synergistic or an antagonistic effect on fructose transport inhibition at both the apical and basolateral side of cells.

## 3. Experimental Section 

### 3.1. Chemicals and Test Materials

[^14^C] Fructose (300 mCi/ mmol) was purchased from American Radiolabeled Chemicals (St. Louis, MO, USA). Dulbecco’s Modified Eagle Medium (DMEM) and all other media supplements were obtained from GIBCO Life Technologies (Gaithersburg, MD, USA). Caco-2 cells were purchased from American Type Culture Collection (Rockville, MD, USA). FK, phloretin, quercetin, catechin, curcumin, demethoxycurcumin and bisdemethoxycurcumin were obtained from Sigma (St. Louis, MO, USA). The nine kinds of culinary plant extract were purchased from the market. Briefly, bayberry bark, chrysanthemum, rosemary and Korean red ginseng were extracted by ethanol/water mixtures; onion, passion flower, touchi and guava leaf were extracted by water; and turmeric was extracted by acetone.

### 3.2. HPLC/MS Analysis

The HPLC system equipped with a photodiode array detector and a Thermo Q Exactive mass spectrometer (Thermofisher Scientific, Bremen, Germany) was employed to determine the chemical signature of the unknown chemical compounds of the two most effective materials. The chromatographic separation was carried out by using a Hypersil C18 column. The mobile phase consisted of water (Solvent A) and 0.02% formic acid in acetonitrile (Solvent B) using a gradient program of 5% Solvent B from 0–1 min, 5%–90% Solvent B from 1–31 min, 90%–5% Solvent B from 31–32 min and then holding at 5% Solvent B for 5 min. The flow rate was 0.3 mL/min. For MS analysis, samples were analyzed in negative ion mode with a spray voltage of 4.2 kV, a sheath/auxiliary gas flow of 35/5, an S-lens voltage of 55 V and a capillary temperature of 320 °C.

### 3.3. Cell Culture

Cells were grown in a humidified incubator (Sanyo, Osaka, Japan) in an atmosphere 5% CO_2_ and 95% air (*v*/*v*; O_2_ partial pressure of 150 Torr) at 37 °C. Caco-2 cells were cultivated in DMEM (25 mM glucose) supplemented with 10% heat-inactivated fetal bovine serum (FBS), 2 mM l-glutamine, 1% nonessential amino acids (NEAA) and 1% penicillin-streptomycin (50 units/mL). The medium was changed every 48 h, and the cells were plated at a density appropriate for each experiment.

### 3.4. Cytotoxicity Test

Cytotoxic effects of culinary plant extracts on Caco-2 cells were measured by the 3-(4,5-dimethylthiazol-2-yl)-2,5-diphenyltetrazolium bromide (MTT) colorimetric assay [21]. Caco-2 cells were seeded on 96-well culture plates at 5 × 10^4^ cells/well and treated with each culinary plant extract for 1 h, and then, 0.5 mg/mL MTT solution were added to each well. After incubation for 4 h at 37 °C, the medium was removed, and the insoluble formazan product was dissolved by DMSO. Absorbance was measured by a microplate spectrophotometer (Spectramax 340-Molecular Devices, Sunnyvale, CA, USA) at 560 nm. Cell viability was calculated as the percentage of viable cells (producing a dark blue formazan product) in the untreated control *versus* treated groups.

### 3.5. Fructose Transport in Cell Systems

To determine [^14^C]-fructose transport inhibition, Caco-2 cells were seeded on 24-well plates and grown to confluence. For the preparation of GLUT5-overexpressing cells and GLUT2 inhibition, Caco-2 cells were cultured in medium containing FK (50 μM) or phloretin (100 μM) and grown to confluence. GLUT5 gene overexpression was confirmed by quantitative RT-RCR in a Step-One-Plus RT-PCR System (Applied Biosystems, Foster City, CA, USA) using the TaqMan method, following extraction of total RNA with TRIZOL (Invitrogen, Carlsbad, CA, USA). The primer sets for target genes were GLUT5 (SLC2A5; Hs00161720_m1). The relative amounts of these mRNAs were normalized to the amount of GAPDH (GAPDH; Hs99999905_m1), and the relative amounts of all RNAs were calculated using the comparative Ct method [22].

Cells were washed twice with PBS and preincubated with Krebs buffer (glucose, 5 mM; HEPES, 30 mM; NaCl, 130 mM; KH_2_PO_4_, 4mM; MgSO_4_, 1 mM; CaCl_2_, 1 mM; pH 7.4) for 30 min at 37 °C. Transport measurement was started by replacing the medium with pre-warmed Krebs buffer without glucose supplemented with 10 mM fructose substrate with [^14^C] fructose and each test sample together for 10 min at 37 °C. The substrate concentration of 10 mM fructose reflected post-prandial circulating concentrations of fructose and the related K_m_ of fructose transporters [7]. Transport was terminated by adding ice-cold PBS, and cells were washed three times with the same solution before lysis with NaOH (0.1 M) in 3-[(3-cholamido-propyl)-dimethylammonio]-1-propanesulfonate (CHAPS, 10 g/L) solution. Aliquots were added to scintillation cocktail for radioactivity determination by the LS 6500 Liquid Scintillation Counter (Beckman Coulter, Fullerton, CA, USA). Aliquots were used for protein measurement using the Bradford assay (Bio-Rad laboratories, Hercules, CA, USA). Radioactive counters per minute (cpm) of [^14^C] fructose were measured for 1 min, and the specific activity was expressed as cpm/mol [5].

### 3.6. Statistical Analysis

Statistical analyses were conducted using SAS (SAS Institute, Cary, NC, USA). All data were expressed as the means ± the standard deviation (SD). Data points were analyzed by nonlinear regression analysis fitting to a 4-parameter logistic curve (SigmaPlot software, SPSS Inc., Chicago, IL, USA), and then, the IC_50_ was calculated for each sample. IC_50_ indicates the concentration at which fructose transport was decreased by half compared to the control. Data were analyzed using the *t*-test with Dunnett’s *post hoc* correction compared to the control. *p* < 0.05 was defined as statistically significant.

## 4. Conclusions 

We demonstrated that guava leaf and turmeric have different signature components, resulting in different effected on apical-facing intestinal fructose transport inhibition in Caco-2 cells. Demethoxycurcumin in turmeric was an important contributor to inhibition of GLUT2-mediated fructose uptake, whereas catechin in guava leaf was the same for GLUT5-mediate fructose uptake. Taken together, these results suggest a possibility that guava leaf and turmeric extracts might be useful to reduce the risks of obesity, diabetes or dyslipidemia from excessive fructose intake.

## Abbreviations

ADP: adenosine diphosphate; AtT20ins cell: GLUT2-overexpressing mouse pituitary adenoma cells; Caco-2: heterogeneous human epithelial colorectal adenocarcinoma cells; FK: forskolin; GLUT: glucose transporter; HFCS: high-fructose corn syrup; HPLC: high performance liquid chromatography; IC_50_: half maximal inhibitory concentration; MS: mass spectrometry; PDA: photodiode array.
